# Annual Meeting of Members

**Published:** 1920-07-03

**Authors:** 


### THE BRITISH HOSPITALS' ASSOCIATION.

### Annual Meeting of Members.

THE Annual Meeting of the Association was held at St. Thomas's Hospital on June 25. The chair, in the first instance, was occupied by Mr. H. Wade Deacon (Chairman of the Council), and supporting him were Lord Winterton and the Hon. Sir Arthur Stanley (Treasurer of St. Thomas's Hospital).

Amongst those present were Mr. E. J. Layton and Mr. T. Hayes (St. Bartholomew's), Mr. F. Neden and Mr. F. Penty (York), Mr. W. H. Head (Cambridge), the Lady Susan Gilmour (Edinburgh), Mr. J. H. Shaw (Stockport), Mr. C. R. W. Offen (Leamington), Mr. C. S. Risbee (Northampton), Mr. R. J. Coles (King's College Hospital), Colonel J. E. Broadbent, C.B. (Reading), Mr. F. Inch (Norwich), Mr. A. Griffiths (Ipswich), Mr. W. Banks (Derby), Mr. G. Cunningham (Middlesex), and Mr. P. Rogers (Poplar).

**The Chairman** apologised for the absence of Lord Sandhurst, who was resigning his Presidency of the Association. He submitted the annual report of the Council, which opens with a reference to the death of two Vice-Presidents—Sir Henry Burdett and Sir William Osler—and Colonel Bruce Vaughan. Sir Henry Burdett, it said, was one of the prime movers in the refounding of the Association in 1910, and for many years took a most active part in the Association's work; his keen personal interest in the administration of the voluntary hospitals was continued right up to, and even during, his last illness, and the effect of his solid work for the voluntary hospitals will last. Sir William Osler's interest in the work of hospitals was as great as his knowledge of disease and its treatment. Colonel Bruce Vaughan was a hospital administrator of wide vision, who showed enormous courage and energy. The Association continued to strengthen its position as representing the voluntary hospitals of the country, and it was hoped that it could soon claim to represent every voluntary hospital in the country. The Chairman said there was a balance in hand of £151 18s. 3d. The expenses had been small, largely because of the amount of voluntary work which had been given. In the future, however, it was likely there must be a larger expenditure for clerical and other assistance.

THE QUESTION OF REGIONAL REPRESENTATION.

**Mr. G. Q. Roberts** (Secretary, St. Thomas's Hospital) proposed the following resolution: "That this meeting approves of the suggestion of the Council that the work of the Association be divided up into sections, that regional committees be instituted throughout the country, and that authority be hereby given to the Council to make such alterations and additions to the existing rules as they may deem necessary."

He said it was well known what were the different natures and circumstances of the hospitals in the various parts of the country, and no one would say the hospitals in London represented those in the various parts of England and Wales; each had its peculiar local circumstances, and if this Association were to be thoroughly representative those must be represented on the Central Council. The differences were clearly evident in the recent negotiations with the Ministry of Pensions, and in the early phases of those negotiations the Ministry had great difficulty in knowing whom to approach as representing all the various hospitals. No other body could approach the claims of this Association in that respect. Representatives in the suggested regions would be asked to concern themselves with their own needs and circumstances, and keep the Central Council informed. Major BASIL RHODES seconded.

**The Chairman**, answering a question by Mr. Griffiths (Ipswich), said the members nominated by each division would be added to the present Council, so that, for a time at least, that body would be somewhat more numerous.

**Major Rhodes** gave a general idea of the suggested division of the kingdom, which it is proposed shall somewhat follow that operating in the case of the Ministry of Pensions.

**The Chairman** said it was hoped that the representative of each division would call a meeting of the hospital representatives in his area to ascertain whether they approved of the idea.

**Lord Winterton** thought it very important that such a scheme for establishing divisions should be operative. The position of the voluntary hospitals, from a political as well as from the financial point of view, was a very precarious one. There was a body of opinion which was in favour of the State taking over the voluntary hospitals. Those present, especially such as came from industrial areas, were aware of the fact that what impressed Parliament and politicians generally was organisation, and if there were a body representing the whole of the interests, whatever they might be, its requests had much greater effect than would any sectional representation. When, during the next two years, the question of what should be the future of the voluntary hospitals should come up, this Association could go to the Government and say, "We represent the whole of the interests of the voluntary hospitals." He deprecated any discussion of details of the regional scheme now; it would be better to confine attention to the broad policy of securing a more thorough and united representation of the whole of the voluntary hospitals of the country. The motion was unanimously carried.

THE RE-ELECTION OF THE COUNCIL.

**Mr. Godfrey Hamilton** proposed the re-election of the Council. He said he noticed that the special hospitals were very poorly represented on the Council, yet they had special claims in such matters as dealing with the Ministry of Pensions; he hoped the Council would bear in mind their particular claims.

**The Right Rev. M. E. C. de Wiart** seconded, and expressed his appreciation, as treasurer of a small hospital in London, of the consideration which small institutions received at the hands of the Council.

**The Chairman** said it was his duty formally to report the resignation of Lord Sandhurst as President of the Association, as his Lordship found himself unable to devote the necessary time to the work. Lord Sandhurst had been helpful to the Association in giving advice from time to time, and the Association was very grateful for all he had done for it.

THE NEW PRESIDENT.

**The Chairman** said he was glad to be able to announce that the Hon. Sir Arthur Stanley had been induced to stand for election to the Presidency. Sir Arthur had from the beginning taken an interest in the affairs of the Association; he constantly attended the meetings of the Council, and his advice and help had been invaluable. He proposed the election of Sir Arthur Stanley as President.

**Mr. J. H. Shaw** (Southport) seconded, remarking that the Association, in accepting the motion, would be in safe hands. The motion was accepted unanimously.

**Sir Arthur Stanley,** on taking the chair, expressed his appreciation of the kind things which had been said about him. He felt a difficulty, as anyone must do, in following Lord Sandhurst, who had been President of the Association during very difficult times. He felt that the chair ought to be occupied by someone who, for his sins, was somewhat in the middle of the hospital movement, for, as Lord Winterton had remarked, the hospitals were in the midst of difficult times, and members of the Association were really in the position of federated employers. All the different interests concerned in hospitals—nurses, doctors, various classes of hospital employees—were well represented; the only class which lacked adequate representation was that of hospital governors and owners. This country's hospitals were, roughly, divisible into two groups, the voluntary and the Poor-Law. It was quite easy to see the latter coming to

**The British Hospitals' Association**—(*continued*).

any agreement, but difficult for the voluntary hospitals, because no body could yet be said thoroughly to represent all the hospitals of the country. It was realised that the time had passed when each voluntary hospital could stand on its own footing. The utmost possible should be done to strengthen the Association, in order that it might have an effective voice in settling what were to be the conditions governing the voluntary hospitals of the country.

**The Chairman** proposed the election of the following honorary officers: Hon. Treasurer, Mr. R. A. Outhwaite; Hon. Solicitor, Sir Perceval Nairne; Hon. Secretary, Mr. J Courtney Buchanan; Hon. Auditor, Sir Arthur Whinney. Mr. Conrad Thies, who was not enjoying very good health, resigned the Hon. Treasurership, to which he had been appointed after his long service as Secretary, and the Association felt very much indebted to him for all he had done.

THE HOURS OF EMPLOYMENT BILL.

**The Chairman** said one of the difficult questions which hospitals had to deal with was that of hours of work for nurses under this Bill. The Ministry of Labour had been in communication with the College of Nursing on the subject, and he understood that they had asked the College of Nursing what organisation represented the governing bodies of the hospitals, and the reply was that this was the Association to apply to. He expected, therefore, that the Ministry of Labour would ask the Association to meet them in order to discuss this question of the hours of employment for nurses. If it could be arranged, he thought it would be very advisable for this Association to have a conference with the representatives of the College of Nursing, in order, if possible, to present to the Ministry of Labour some definite agreed scheme. If that were done, he had reason to think the Ministry of Labour would insert a provision for their scheme in the Bill. Two or three representatives would be needed at easy call, and he suggested the names of Mr. Leo Lyle, M.P., who had been very active in connection with the Bill for the State Registration of Nurses, the Rev. G. B. Cronshaw, and Mr. G. Q. Roberts, St. Thomas's Hospital. He suggested such a small committee should be authorised to meet the Council of the College of Nursing. Mr. HAZELL (Manchester) seconded.

During a short discussion it was pointed out that there was not only the increased wages bill to be considered, but additional accommodation for the extra nurses; also that the small hospitals should be represented, for their conditions, being less strenuous than those in large cities, were different.

**The Chairman** pointed out that the work of such a small committee would only be of an advisory nature; it could not bind the Association to anything, but must report to the Association.

Mr. Hazell's name was added to the committee, and then the names were approved.

**The Chairman** thought the conclusions of the committee ought to be circulated to all the members, with a request for comments thereon.

### The Hospitals' Position and Outlook.

**The Chairman** said that all hospitals were going through bad times, and this year was perhaps the worst. But the King Edward Fund had given £250,000 for London hospitals. The actual requirements of the London hospitals to be met must include extraordinary expenditure, which had not been very great during those five years, for everybody had put off everything they could in the way of renewals; but that expenditure would now have to be incurred, and it was one of the most difficult things to be faced. The ordinary expenditure of hospitals outside London had nearly doubled since 1914; on the other hand, the ordinary income had expanded by over one-third. In that must be included much money which had been received from the War Office in capitation grants. The gratifying feature was that in the year 1919, when the grants from the War Office were very much smaller because there were fewer of the military undergoing treatment, there was an appreciable rise in the ordinary income, showing that the country was beginning to respond to some extent to the increased needs of the hospitals. With regard to the London Hospitals, he did not doubt there was in them a very large scope for effort. Under 8 per cent. of the population of London subscr[illegible] to the hospitals, and if about 20 per cent. of them could be induced to do so there must be a very good result. Another matter, which was decidedly a part of their scheme, was that for work they did for the Government they should be adequately paid. The Government had—and quite rightly—taken up the fight against tuberculosis, against venereal disease, and so on; and if the hospitals could not find buildings for the work the Government would themselves have to provide them, at enormous cost. Hence, if hospitals were to join in that work they must be paid adequately—not the small amount received now, but the full cost of management. A great difficulty to be encountered was in meeting the increased costs of the hospitals and at the same time to try and find some way of paying off the debts pressing upon them. If that could be done on a sufficient scale, then not only would their deficits on maintenance be paid off, but there would also be a substantial sum over for the repairs and renewals they all had to face. If they could get the money, then he would be very confident about the continuance of the voluntary system; if they did not get it, then they would all have to work harder. Nevertheless, he felt there was a strong wish on the part of the public to do what they could to save hospitals from going on the rates, and there was a very distinct wish on the part of the Ministry of Health that they should be saved from having to go to the Exchequer.

**Major Lovelock** said there were many districts in which money had been raised through having large hospitals as war memorials. The hospital with which he was connected had a certain amount of money, but if the Government wished for *x*-ray equipments and other specialities in connection with the hospital's work, then it seemed to him they ought to pay for such things. Would it be prejudicing the position of hospitals generally if a definite attempt were made to get the Government to give grants towards the special features mentioned in the report as necessary, more particularly in country districts?

The CHAIRMAN, in reply, said he was merely expressing his own opinion when he said that in connection with extensions, extra beds, or other requirements it would be quite a fair thing to ask the Government for a grant towards the sum needed for such purposes. There were, however, many reasons against asking the Government for an annual subsidy for hospitals, for it must eventually mean their coming in and taking over the hospital in question. After all, it was not the hospitals they were looking after, but the health of the people, and where extensions or other requirements were necessary for the betterment of the health of the people, then it was, in his opinion, a very fair subject for a Government grant.

A vote of thanks to the Chairman terminated the proceedings.

